# Monoamine oxidase binding not expected to significantly affect [^18^F]flortaucipir PET interpretation

**DOI:** 10.1007/s00259-022-05822-9

**Published:** 2022-05-21

**Authors:** Justin P. Wright, Jason R. Goodman, Yin-Guo Lin, Brian P. Lieberman, Jennifer Clemens, Luis F. Gomez, Qianwa Liang, Adam T. Hoye, Michael J. Pontecorvo, Kelly A. Conway

**Affiliations:** Avid Radiopharmaceuticals, Eli Lilly & Company, Philadelphia, PA USA

**Keywords:** Flortaucipir, PET, Alzheimer’s disease, Monoamine oxidase, Tau neurofibrillary tangles

## Abstract

**Purpose:**

[^18^F]-labeled positron emission tomography (PET) radioligands permit *in vivo* assessment of Alzheimer’s disease biomarkers, including aggregated neurofibrillary tau (NFT) with [^18^F]flortaucipir. Due to structural similarities of flortaucipir with some monoamine oxidase A (MAO-A) inhibitors, this study aimed to evaluate flortaucipir binding to MAO-A and MAO-B and any potential impact on PET interpretation.

**Methods:**

[^18^F]Flortaucipir autoradiography was performed on frozen human brain tissue slices, and PET imaging was conducted in rats. Dissociation constants were determined by saturation binding, association and dissociation rates were measured by kinetic binding experiments, and IC_50_ values were determined by competition binding*.*

**Results:**

Under stringent wash conditions, specific [^18^F]flortaucipir binding was observed on tau NFT-rich Alzheimer’s disease tissue and not control tissue. *In vivo* PET experiments in rats revealed no evidence of [^18^F]flortaucipir binding to MAO-A; pre-treatment with MAO inhibitor pargyline did not impact uptake or wash-out of [^18^F]flortaucipir. [^18^F]Flortaucipir bound with low nanomolar affinity to human MAO-A in a microsomal preparation *in vitro* but with a fast dissociation rate relative to MAO-A ligand fluoroethyl-harmol, consistent with no observed *in vivo* binding in rats of [^18^F]flortaucipir to MAO-A. Direct binding of flortaucipir to human MAO-B was not detected in a microsomal preparation. A high concentration of flortaucipir (IC_50_ of 1.3 μM) was found to block binding of the MAO-B ligand safinamide to MAO-B on microsomes suggesting that, at micromolar concentrations, flortaucipir weakly binds to MAO-B *in vitro*.

**Conclusion:**

These data suggest neither MAO-A nor MAO-B binding will contribute significantly to the PET signal in cortical target areas relevant to the interpretation of [^18^F]flortaucipir.

**Supplementary Information:**

The online version contains supplementary material available at 10.1007/s00259-022-05822-9.

## Introduction

Alzheimer’s disease (AD) is characterized by misfolded protein aggregates, specifically extracellular amyloid-β plaques [1, 2], and hyperphosphorylated tau neurofibrillary tangles (NFTs) [3]. Historically, AD has been diagnosed only by neuropsychological testing during the dementia stage and confirmed by autopsy [4–6]. Consequently, some patients have been misdiagnosed (found to be lacking the hallmark amyloid-β plaques and tau NFTs at autopsy) and as a result received inappropriate medical treatment [7]. With improvements in biomarker detection, particularly by positron emission tomography (PET), we can now utilize non-invasive imaging techniques to visualize the hallmark pathologies of AD in living patients [8–10]. [^18^F]-labeled PET radioligands that bind and visualize amyloid-β plaques have been approved for several years [11–13]. [^18^F]Flortaucipir, which binds to paired helical filament (PHF) tau in NFTs with high affinity [14, 15], became the first PET tracer approved to estimate the density and distribution of tau NFTs. PET images with [^18^F]flortaucipir accurately reflect tau NFTs after post-mortem pathological examination [16, 17].

[^18^F]Flortaucipir is structurally similar to known reversible inhibitors of monoamine oxidase A (MAO-A), including harmine and fluoroethyl-harmol. Off-target binding has been noted in areas of the brain where aggregated tau is unexpected, and there have been conflicting reports in the literature about possible MAO-A and MAO-B binding relationships with [^18^F]flortaucipir [18–20]. Furthermore, PET signal for the putative tau tracer [^18^F]THK5351 has been reported to be greatly reduced following treatment with the MAO-B inhibitor selegiline (deprenyl) [21]*.* The present study aimed to investigate and characterize any binding relationship between [^18^F]flortaucipir and MAO-A or MAO-B *in vivo* using PET imaging and by *in vitro* competition and saturation binding experiments.

## Materials and methods

### Radiosynthesis of [^18^F]flortaucipir and [^18^F]fluoroethyl-harmol

[^18^F]Flortaucipir was produced as described previously [22]. [^18^F]Fluoroethyl-harmol was produced following the procedure as described by Schieferstein et al. [23].

### [^18^F]Flortaucipir autoradiography: comparison of stringent and mild wash conditions

Autoradiography was performed on frozen human brain slices from AD donors (*N* = 3, PHF tau-rich as determined by AT8 immunohistochemistry) and age-matched non-AD control donors (*N* = 6, devoid of tau NFTs and beta-amyloid neuritic plaques as determined by immunohistochemistry). Brains slices were obtained from the National Disease Research Interchange (Philadelphia, PA).

In brief, 500 μL of [^18^F]flortaucipir (0.74 MBq [20 μCi]) in 2.5% dimethyl sulfoxide (DMSO)/2.5% ethanol (EtOH)/phosphate-buffered saline (PBS), pH 7.4, was pipetted onto each slide. Adjacent sections were incubated with [^18^F]flortaucipir and non-radioactive flortaucipir (1 μM). After 60-min incubation at room temperature, the tissue was washed under mild (4 × 2 min in PBS) or stringent wash conditions (4 × 2 min each in PBS, 30% EtOH/PBS, 70% EtOH/PBS, and PBS). After drying under ambient conditions, the sections were placed in a cassette, exposed to a phosphor imaging plate overnight, and then scanned with a GE Typhoon FLA7000 Bio-Imaging System.

### [^18^F]Flortaucipir autoradiography: competition binding assay with MAO-A and MAO-B inhibitors

Autoradiography competition binding assay of [^18^F]flortaucipir binding to normal human brain was used to determine the IC_50_ values of flortaucipir, MAO-A inhibitors clorgyline (Sigma-Aldrich) and fluoroethyl-harmol (prepared by alkylation of harmol [Alfa Aesar] with 1-bromo-2-fluoroethane as described by Ng et al. [21]), and MAO-B inhibitors deprenyl (Sigma-Aldrich) and safinamide (TCI). Adjacent frozen sections (10 μm thick) from a normal human temporal cortex (sourced from National Disease Research Interchange) were incubated with a fixed concentration of [^18^F]flortaucipir (0.74MBq [20 μCi] in 500 μL, 1.4 nM each slide) with varying concentrations of competing compound (log serial dilution from 10 μM to 0.001 μM) in 2.5% DMSO/2.5% EtOH/PBS, pH 7.4. After 60-min incubation at room temperature, PBS washes (4 × 2 min) were used to remove any unbound ligand. After drying under ambient conditions, the sections were placed in a cassette and exposed to a phosphor imaging plate overnight. The plate was then scanned with a GE Typhoon FLA7000 Bio-Imaging System. Signal intensity (counts/pixel) on each section was measured using Multi Gauge V3.0 Imaging software. Total binding was evaluated by nonlinear regression using GraphPad Prism v8 to determine IC_50_ values.

### *In vivo* PET imaging

All procedures in this study followed the guidelines of the Institutional Animal Care and Use Committee of the University of the Sciences, Philadelphia, PA. A Siemens Inveon Multimodality Scanner was used for micro-PET/computed tomography (CT) imaging. Male wild-type Sprague-Dawley rats (128 ± 58 g) were anesthetized with 3% isoflurane with oxygen. Rats were administered [^18^F]fluoroethyl-harmol or [^18^F]flortaucipir via tail vein injection (7.4-14.8MBq [200–400 μCi] in a total volume of 200 μL). For blocking studies, rats were pre-treated with pargyline (Sigma-Aldrich, 50 mg/kg i.p.) 30 min prior to scans; control rats were pre-treated with 0.9% saline injection (Hospira, i.p.). A short high-resolution CT scan was first conducted for anatomical registration, followed by a 120-min dynamic PET scan. PET images were generated for each minute of the acquisition time. Uptake of the tracers was determined by visually drawing regions of interest based on the fused PET/CT images, and corresponding activity values were determined using Inveon Research Workplace software. All values are represented as % injected dose per gram (ID/g). A total of 6 rats were studied (3 control, 3 pre-treated) for [^18^F]fluoroethyl-harmol and 8 rats for [^18^F]flortaucipir (4 control, 4 pre-treated).

### PHF preparation

Purified, soluble PHF was isolated from AD brain tissue using a protocol modified from the procedure described by Jicha et al. [24]. Briefly, homogenized AD cortex underwent high pressure batch–gas expansion using a Parr Cell disruption bomb and centrifuged at 28 kg. Soluble PHF was isolated from the supernatant by affinity chromatography using an MC1-Affigel 10 column (25 mL flow rate), with a high guard column (4 cm) of Sepharose 400 Superflow, recycling supernatant twice through the column over 18–20 h at 4 °C. Bound PHF was eluted with two column volumes of 3M potassium thiocyanate. Isolated PHF was analyzed in a tau MC-1 ELISA and a tau AT8 ELISA, which recognizes tau phosphorylated at both Ser 202 and Thr 205 [3].

### K_d_ and IC_50_ determination

The dissociation constant (K_d_) was determined by saturation binding, in which the total and non-specific binding of the radioligand was measured at various concentrations. Human recombinant MAO-A, MAO-B, and control microsomes were acquired from Sigma (M7316, M7441, and M7566, respectively). All microsome preparations were at a concentration of 5 mg protein/mL. The reaction mixture (250 μL) contained protein target and ^18^F-radioligand, serially diluted in PBS, pH 7.4; assays were performed in PBS (0.01% bovine serum albumin [BSA]) in 96-well polypropylene microplates. Non-specific binding (NSB) was defined as radioligand binding in the absence of target or in the presence of an excess of non-radioactive compound selective for the target: T808 (10 μM, synthesized at Avid Radiopharmaceuticals according to published procedures) [25] for PHF, clorgyline (1–10 μM) for MAO-A, and deprenyl (1–10 μM) for MAO-B. After incubation (1.5 h at 37 °C), the bound radioactivity was harvested onto Millipore MultiScreen®_HTS_ FB filter plates by vacuum filtration (Millipore MultiScreen®_HTS_ Vacuum Manifold), followed by 5 PBS washes. Filters were assayed for radioactivity in a Wizard 2480 automatic gamma-counter (Perkin Elmer). Data were analyzed by nonlinear regression analysis (GraphPad Prism) to determine K_d_ for the radioligand.

For non-radioactive compounds, K_d_ was determined by saturation binding in 96-well polypropylene microplates; the reaction mixture (250 μL) contained a fixed amount of protein and 12 concentrations of ligand and PBS (0.02% BSA). After incubation (1.5 h at 37 °C), bound ligand was harvested by vacuum filtration onto a glass fiber filter plate (Pall® Acroprep™ Advance 96-well 1.0 μm), using a Millipore MultiScreen®_HTS_ Vacuum Manifold, followed by 5 PBS washes. Bound ligand was then eluted into a 96-deepwell 700 μL polypropylene plate with 250 μL internal standard in methanol by centrifugation at 4000 RPM for 5 min. Each sample (15 μL) was analyzed via liquid chromatography-mass spectrometry (LC-MS, see supplementary information for more detail). Data were analyzed by nonlinear regression analysis (GraphPad Prism).

IC_50_ values for each compound were determined by competition binding. The reaction mixture (250 μL) contained a set amount of protein and ligand as well as a decreasing amount of inhibitor, from 10 μM to 0.32 nM (1/2 log dilution series), in PBS containing 0.02% BSA in 96-well polypropylene microplates. After incubation for 1.5 h at 37 °C, bound ligand was harvested and analyzed by LC-MS as described above. IC_50_ values were determined by nonlinear regression analysis using GraphPad Prism.

### Kinetic binding study

The association (k_on_) and dissociation (k_off_) rates were determined by kinetic binding experiments in which binding of a radioligand to a target (PHF tau or MAO-A or MAO-B microsomal preparations) was measured at multiple timepoints. The reaction mixture (250 μL) contained protein target and ^18^F-radioligand (1 nM); assays were performed in PBS containing 0.01% BSA in 96-well polypropylene microplates. At each timepoint of the 37 °C incubation, the bound radioactivity was harvested by vacuum filtration onto Millipore MultiScreen®_HTS_ FB filter plates, using a Millipore MultiScreen®_HTS_ Vacuum Manifold, followed by 5 PBS washes. Once the equilibrium signal was reached, dissociation was initiated by adding excess non-radioactive compound. Measurements continued until no binding was detected. Filters containing bound [^18^F]flortaucipir were assayed and analyzed for radioactivity as previously noted.

## Results

**Fig. 1:**
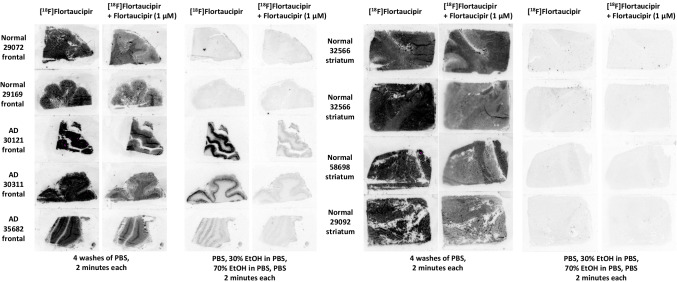
[^18^F]Flortaucipir autoradiography on AD and normal tissues. Comparison of stringent and mild wash conditions on post-mortem brain sections (frontal cortex and striatum). Under stringent washing conditions, binding observed in tau NFT-rich AD tissue and not normal control. Abbreviations: AD, Alzheimer’s disease; EtOH, ethanol; PBS, phosphate-buffered saline

[^18^F]Flortaucipir autoradiography was performed on frontal cortex and striatum frozen human brain tissue slices from multiple AD and control donors to assess tau NFT and off-target binding (Fig. 1). Under stringent washing conditions (30% and 70% EtOH), [^18^F]flortaucipir binding was observed only on tau NFT-rich AD tissue, not on control tissue. Autoradiography signal on AD tissue was found to be saturable in the presence of non-radioactive flortaucipir. However, when mild washing conditions were used, binding of [^18^F]flortaucipir was observed on both AD and control tissues, in an off-target pattern different than that observed using stringent washing conditions, and for which binding was only partially saturable by 1 μM flortaucipir.

**Fig. 2:**
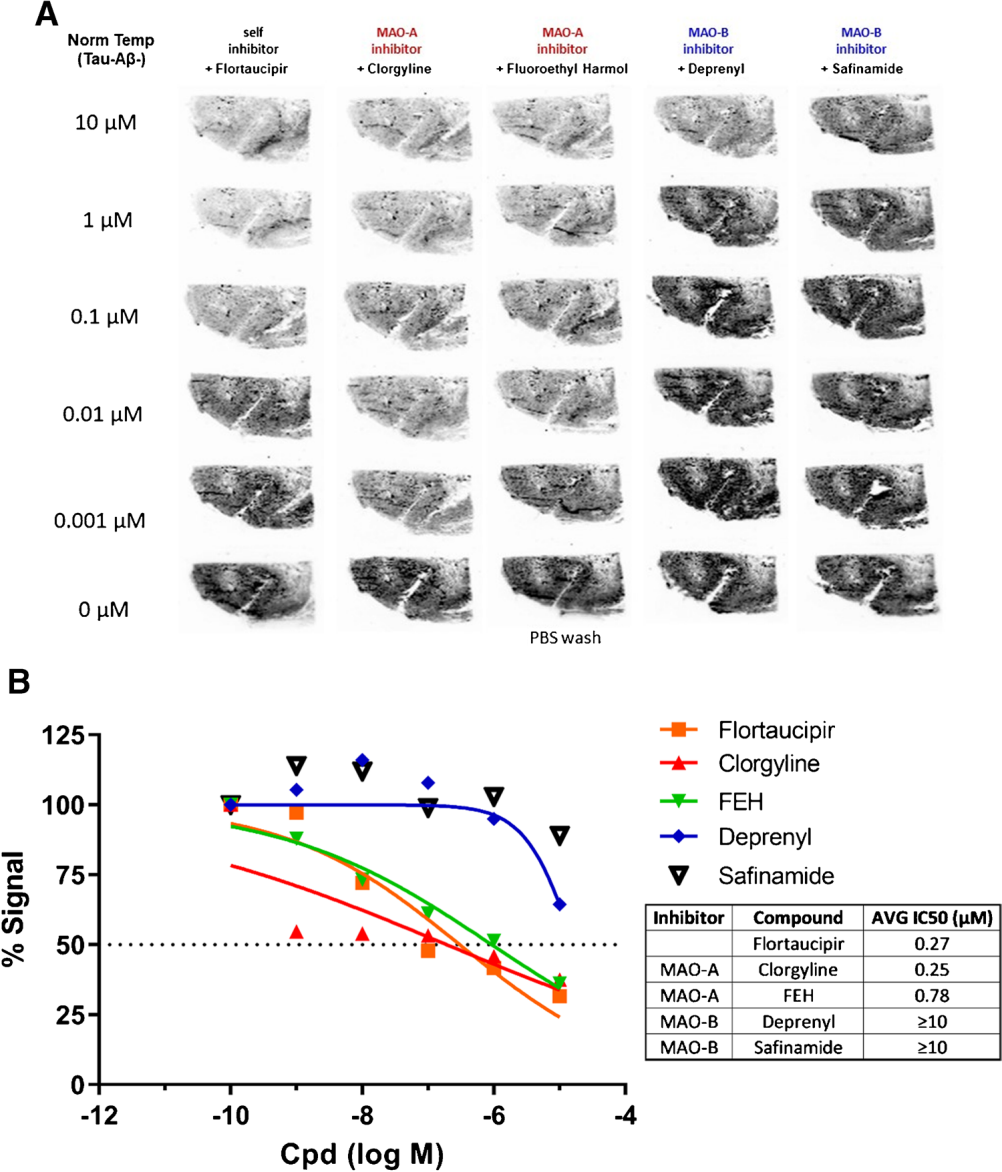
Flortaucipir and MAO-A/B inhibitor competition binding. [^18^F]flortaucipir autoradiography on normal (tau NFT negative) human brain tissue (**A**). MAO-B ligands only weakly blocked off-target binding of [^18^F]flortaucipir on normal tissues (IC_50_ ≥ 10 μM, curve fit by nonlinear regression) (**B**). Abbreviations: AVG, average; Cpd, compound; FEH, fluoroethyl-harmol; MAO, monoamine oxidase; PBS, phosphate-buffered saline

To further understand the off-target binding observed under mild wash conditions, inhibition of [^18^F]flortaucipir binding by MAO-A or MAO-B inhibitors was evaluated on temporal cortex brain sections from control subjects, devoid of tau NFTs, and beta-amyloid neuritic plaques (Fig. 2A, 2B). Under essentially the same mild washing conditions, non-radioactive flortaucipir and MAO-A inhibitors clorgyline and fluoroethyl-harmol weakly blocked [^18^F]flortaucipir binding similarly, with IC_50_ values of 0.27 μM, 0.25 μM, and 0.78 μM, respectively. Binding of [^18^F]flortaucipir was only very weakly blocked by MAO-B inhibitors deprenyl and safinamide (IC_50_ ≥ 10 μM). It is noted that only approximately 50% of the [^18^F]flortaucipir bound to normal brain sections under mild wash conditions was displaced, while the remainder was non-specific and non-saturable since it could not be displaced by high levels of MAO-A ligand or flortaucipir itself.

**Fig. 3:**
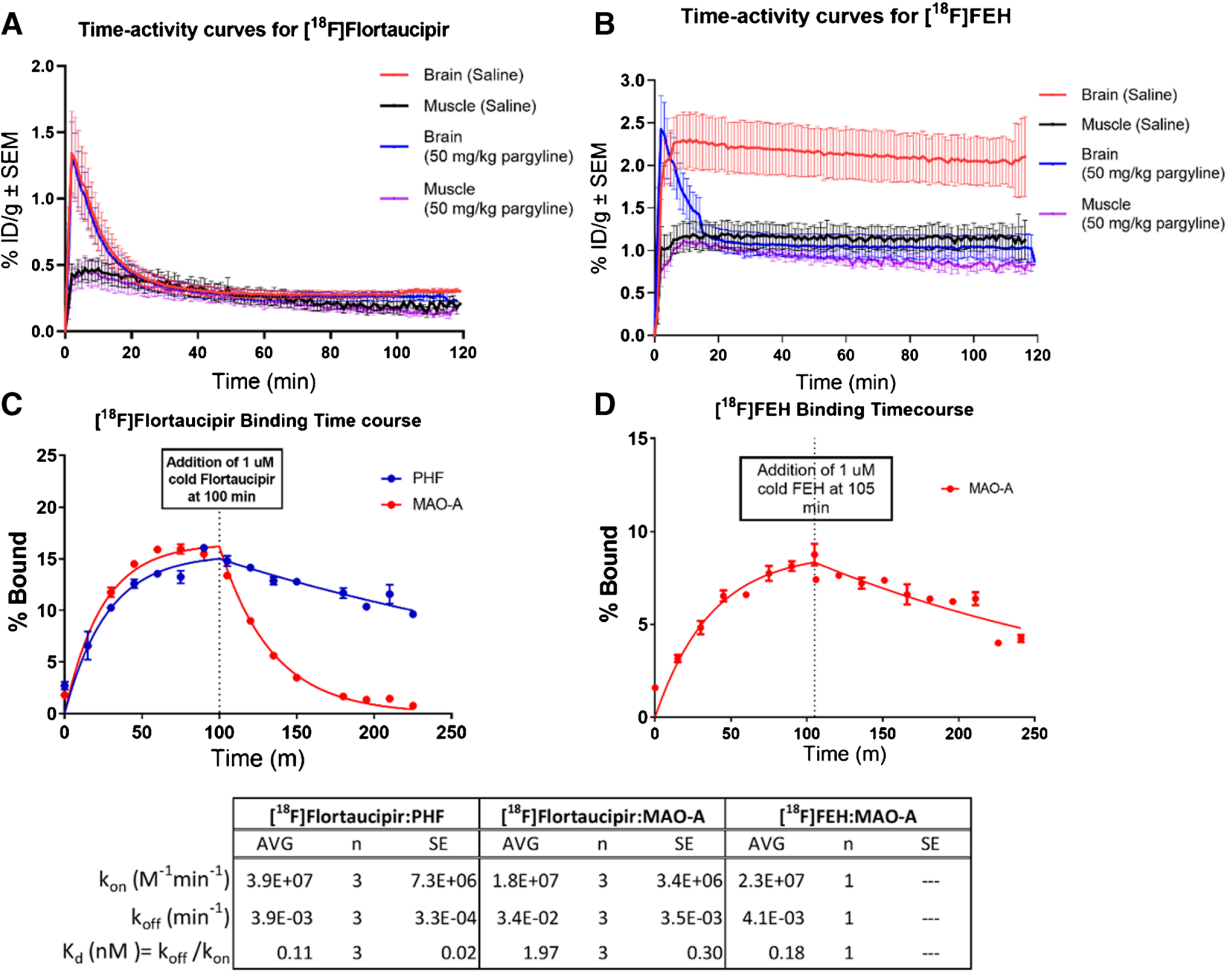
PET time activity curves and binding kinetics of [^18^F]flortaucipir to MAO-A, compared with MAO-A ligand [^18^F]FEH. PET time activity curves were generated for [^18^F]flortaucipir for rats pre-treated with saline and pargyline (50 mg/kg) (**A**; *n* = 4 per group) and for [^18^F]-FEH (fluoroethyl-harmol) for rats pre-treated with saline and pargyline (50 mg/kg) (**B**; *n* = 3 per group). Data are mean ± SEM. Kinetic binding curves were generated for [^18^F]flortaucipir binding to PHF and MAO-A (**C**) and for [^18^F]-FEH (fluoroethyl-harmol) binding to MAO-A (**D**). Data are mean ± SD. Abbreviations: AVG, average; MAO, monoamine oxidase; n, number of replicates; PET, positron emission tomography; PHF, paired helical filament; SD, standard deviation; SEM, standard error of mean

PET studies were conducted in rats to compare retention of a selective and reversible MAO-A tracer, [^18^F]fluoroethyl-harmol, to that of [^18^F]flortaucipir in both control and 50 mg/kg pargyline (an irreversible MAO-A/B inhibitor) pre-treated rats (Fig. 3A, 3B). In control rats, [^18^F]fluoroethyl-harmol crossed the blood-brain barrier, reached a peak of 2.3 %ID/g at 2 min, and was retained in the brain throughout the scan period with 2.1 %ID/g still present at 110 min, consistent with its strong binding relationship with MAO-A. However, for [^18^F]fluoroethyl-harmol in the pargyline pre-treated group, peak brain activity reached 2.4 %ID/g at 2 min, followed by a rapid washout to 1.0 %ID/g by 20 min. In contrast, [^18^F]flortaucipir displayed nearly identical brain uptake with rapid clearance in both the control and pargyline groups (Fig. 3B) consistent with a minimal *in vivo* MAO-A PET signal. Results shown in the Supplemental Information, Fig. S1, further confirm the presence of MAO-A binding sites in rats and explore the interaction of flortaucipir with these sites. [^18^F]flortaucipir autoradiography on rat brain slices using mild (aqueous) wash conditions demonstrated binding of [^18^F]flortaucipir that could be blocked by excess non-radioactive flortaucipir, the MAO-A inhibitor clorgyline and the MAO-A/B inhibitor pargyline but not by the MAO-B inhibitor deprenyl. These results are consistent with the binding pattern observed on human brain tissue using mild washing conditions in Fig. 2.

The K_d_ for binding of [^18^F]flortaucipir to isolated human PHF was determined to be 0.57 nM compared to 2.0 nM to recombinant MAO-A. The on/off rates (k_on_/k_off_) for [^18^F]flortaucipir binding to PHF tau and MAO-A (Fig. 3C) were measured (under mild washing conditions) using [^18^F]fluoroethyl-harmol as a positive control for MAO-A binding (Fig. 3D). While similar association rates (k_on_) were observed for each ligand-protein pair (1.8E07–3.9E07 M^−1^min^−1^), the dissociation rate (k_off_) for [^18^F]flortaucipir:MAO-A was found to be approximately 8–9 times faster than that of [^18^F]fluoroethyl-harmol:MAO-A and [^18^F]flortaucipir:PHF.

**Fig. 4:**
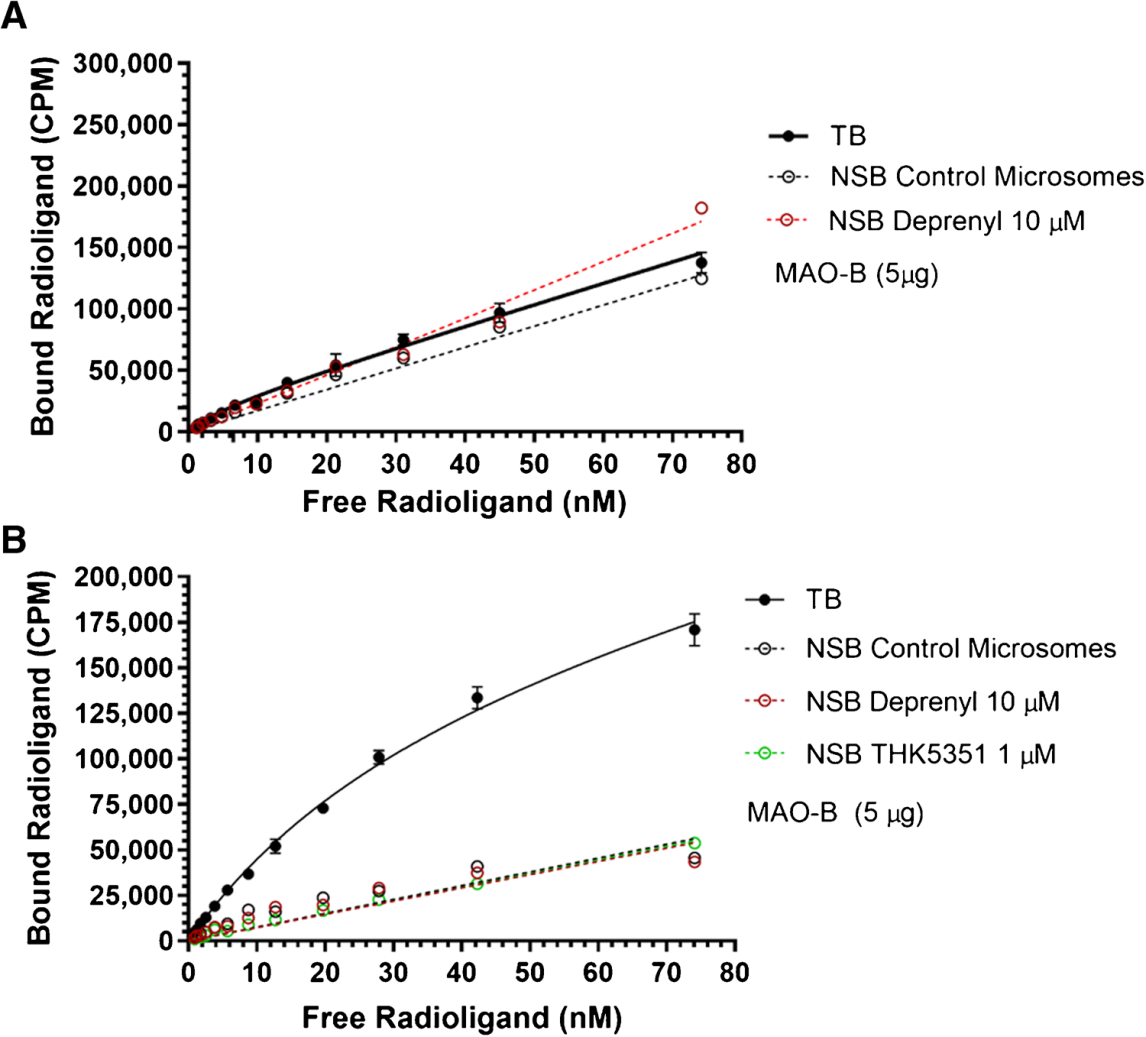
Comparison of *in vitro* binding of [^18^F]flortaucipir to MAO-B compared to [^18^F]THK5351. Saturation binding [^18^F]flortaucipir (**A**) and [^18^F]THK5351 (**B**) to MAO-B microsomes in the presence of competing non-radioactive compound (10 μM deprenyl or 1 μM THK5351) or absence of target (control microsome). TB and NSB fit by nonlinear regression. Data are mean ± SD. Abbreviations: CPM, counts per minute; MAO, monoamine oxidase; NSB, non-specific binding; TB, total binding

Studies using the putative tau tracer [^18^F]THK5351 have shown that its affinity toward MAO-B confounds its quantification of tau by PET [21]*.* To explore the potential affinity of flortaucipir for MAO-B, *in vitro* saturation binding of [^18^F]flortaucipir and [^18^F]THK5351 to MAO-B microsomes was conducted (Fig. 4). The K_d_ for the binding of [^18^F]THK5351 to recombinant MAO-B was determined to be 37 ± 1.8 nM with a B_max_ of 49 ± 6.3 pmol ligand/mg total protein, when NSB was defined as binding to control microsomes (absence of target). Similar K_d_ and B_max_ were determined when 10 μM deprenyl (K_d_ of 39 ± 1.1 nM; B_max_ of 51 ± 5.5 pmol ligand/mg total protein) or 1 μM THK5351 (K_d_ of 40 ± 0.7 nM; B_max_ of 54 ± 3.4 pmol ligand/mg total protein) were used to define NSB. In contrast, no specific binding of [^18^F]flortaucipir to MAO-B was detected using the same assay conditions.

**Fig. 5:**
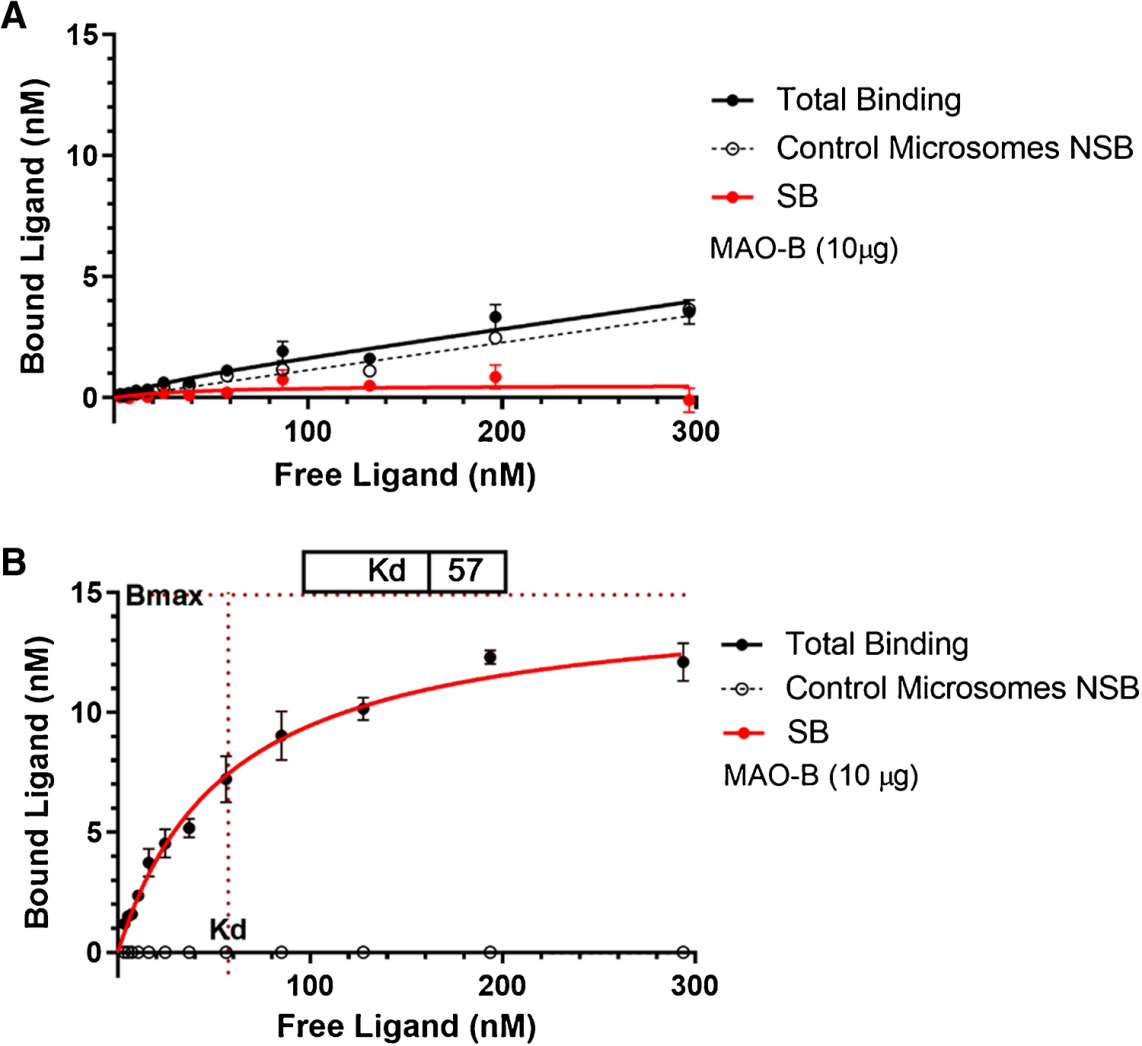
Saturation binding isotherm of flortaucipir (**A**) and safinamide (**B**) to MAO-B. Total binding (TB): binding of ligand, specific, and non-specific. Non-specific binding (NSB): binding of ligand in presence of competing non-radioactive compound (10 μM deprenyl) or absence of target (control microsome). Specific binding (SB): specific binding to MAO-B microsomes; total binding minus non-specific binding. TB and NSB fit by nonlinear regression using GraphPad Prism. Data are mean ± SD. Abbreviations: MAO, monoamine oxidase; SD, standard deviation

Additional saturation binding experiments were conducted at higher flortaucipir concentrations in a liquid chromatography-mass spectrometry (LC-MS) direct binding assay (Fig. 5). Again, the K_d_ for flortaucipir binding to recombinant MAO-B microsomes could not be determined; no specific binding was observed as evidenced by the lack of separation between total binding and non-specific binding curves. By comparison, the K_d_ for the binding of safinamide, a selective and reversible MAO-B ligand, to recombinant MAO-B was 57 nM with a B_max_ of 446 pmol ligand/mg total protein when binding to control microsome (absence of target) is used to define NSB.

**Fig. 6:**
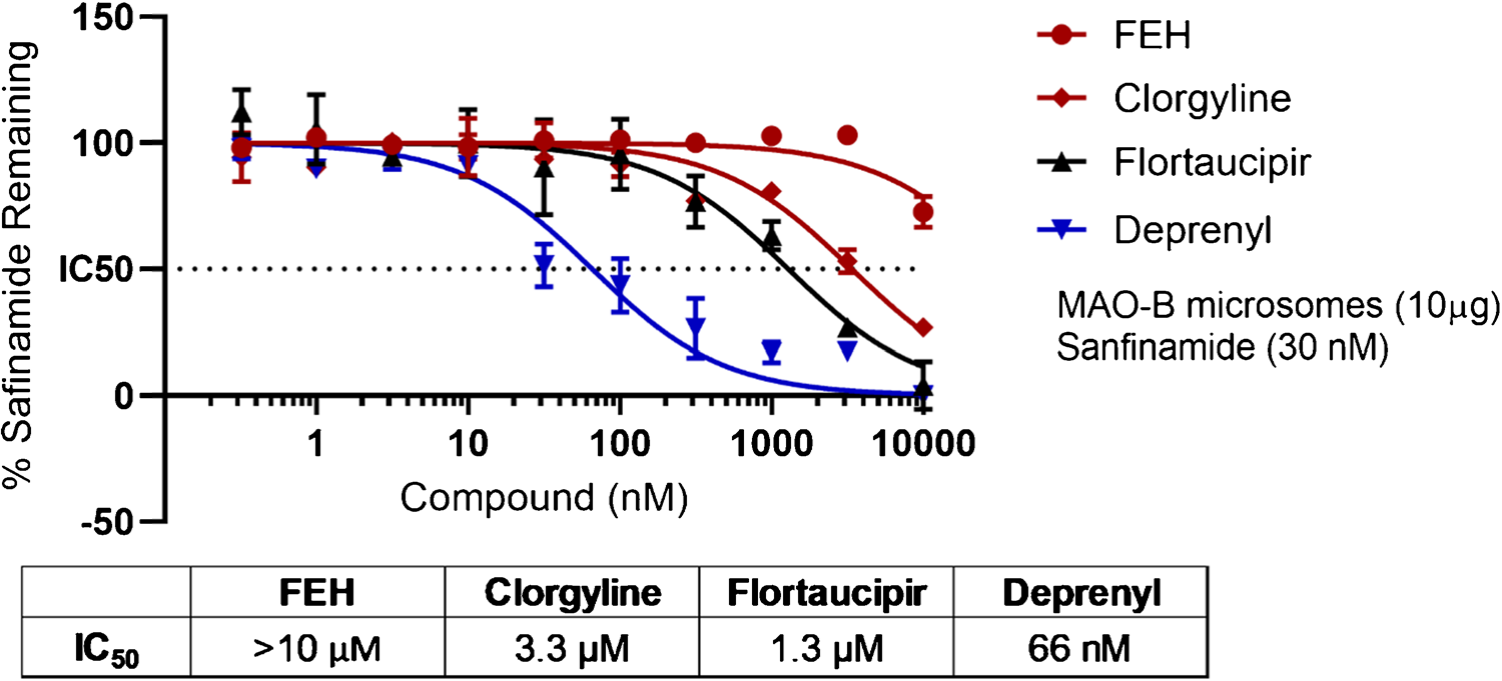
IC_50_, displacement of safinamide bound to MAO-B microsomes. Safinamide binding at a concentration of 30 nM to 10 μg MAO-B microsome was measured in the presence of fluoroethyl-harmol (FEH), clorgyline, flortaucipir, and deprenyl. Data are mean ± SD. Abbreviations: MAO, monoamine oxidase; SD, standard deviation

Finally, safinamide binding at a concentration of 30 nM to 10 μg MAO-B microsomes was measured by LC-MS in the presence of deprenyl, flortaucipir, clorgyline, and fluoroethyl-harmol (Fig. 6). The MAO-B inhibitor deprenyl displaced safinamide with an IC_50_ of 66 nM. Flortaucipir and MAO-A inhibitor clorgyline both weakly inhibited safinamide binding to MAO-B, with IC_50_ of 1.3 μM and 3.3 μM, respectively. MAO-A inhibitor fluoroethyl-harmol showed weak competition against safinamide to MAO-B with an indeterminable IC_50_ (> 10 μM).

## Discussion

This study aimed to characterize any binding relationship of [^18^F]flortaucipir with MAO-A or MAO-B and evaluate if this would impact the interpretation of [^18^F]flortaucipir PET. We observed binding of [^18^F]flortaucipir to MAO-A (2.0 nM K_d_) in a microsomal preparation *in vitro*, however with a fast dissociation rate relative to that of [^18^F]fluoroethyl-harmol:MAO-A and [^18^F]flortaucipir:PHF, consistent with the lack of binding in our *in vivo* experiment. Direct binding of flortaucipir to MAO-B was not detected *in vitro*, but a high concentration of flortaucipir (IC_50_ of 1.3 μM) was found to block safinamide binding to MAO-B in microsomes suggesting that, at micromolar concentrations, flortaucipir weakly binds to MAO-B *in vitro*.

The affinity and specificity of flortaucipir have been well characterized with [^18^F]flortaucipir PET images in AD patients showing a pattern of distribution consistent with the pathological observations of Braak [26] and others for the distribution of tau NFTs in AD brains [14–17]. However, localization of [^18^F]flortaucipir is seen also in striatum and choroid plexus in both AD patients and those with no evidence of disease [27]. It is possible that some of the signal in the choroid plexus can spill over into adjacent regions [27, 28]. The source of this choroid plexus binding is not known but may reflect binding to iron/calcification or, less likely, Biondi bodies [29, 30]. Presumed off-target elevations of PET signal for multiple tau PET tracers, including [^18^F]flortaucipir, [^18^F]MK-6240, and [^18^F]THK5351, have also been observed in structures rich in neuromelanin and melanin-containing cells [29, 31–34]. These regions, however, reside outside of the AD-associated neocortical areas examined for visual interpretation, and thus, the observed activity has little or no impact on ability to visually interpret flortaucipir AD PET scans. The causes of the off-target binding in striatum and choroid plexus detected by [^18^F]flortaucipir PET have not been definitively identified; multiple labs have been unable to detect the same pattern of off-target binding observed in [^18^F]flortaucipir PET by autoradiography studies on post-mortem tissue [31, 35, 36]. However, none of the aforementioned potential off-target binding sites have the same potential to be confused with tau deposition in AD as would binding to MAO-B in activated astrocytes.

Several other tau tracers in development have exhibited off-target binding presumably due to interaction with MAO-B [21, 37, 38]. This has raised the question whether [^18^F]flortaucipir also interacts with MAO-B; thus far, conflicting evidence has failed to clarify any possible interaction [20, 38–40]. [^18^F]THK5351, a radiotracer that has been investigated as a possible tau imaging agent, is known to bind to both NFT tau and MAO-B [41, 42], and MAO-B inhibitors rasagiline and selegiline (deprenyl) significantly attenuate the uptake of [^18^F]THK5351 in humans [21, 43]. MAO-B interaction could impact the interpretation of PET scans for tau-targeted radioligands due to signal overlap, as MAO-B is expressed by reactive astrocytes that often co-localize with tau NFTs [44–48].

Concerns about off-target binding associations to MAO-B or MAO-A have prompted [^18^F]flortaucipir PET studies in non-human primates to elucidate potential binding to MAOs [18, 49]. We do not find the data in these studies to be consistent with specific *in vivo* binding and retention of flortaucipir to MAOs or specific displacement of flortaucipir binding by known MAO inhibitors in any brain region. Drake et al. [18] acknowledged that in their study, flortaucipir distributed non-specifically in the NHP brain; the washout curves for hippocampus, cerebellum, thalamus, basal ganglia, and cortex appear indistinguishable from each other (as shown in Drake et al. [18] Supplemental Information, Fig. S5). This pattern of distribution is in contrast to reported MAO-B ligands such as [^11^C]deprenyl-d_2_ [50] and [^18^F]FSL25.1188 [51], which demonstrated high signal in both thalamus and basal ganglia, and reduced retention in the cerebellum or MAO-A ligands such as [^11^C]harmine [52], which demonstrate higher activity in areas such as the thalamus and putamen relative to the cerebellum. In these three studies [50–52], activity in areas of high MAO expression such as thalamus and basal ganglia is significantly higher (1.5- to 2-fold) than in cerebellum, an area of low MAO expression [53], whereas a similar pattern is not observed in the study by Drake et al. In the non-human primate study by Hostetler et al. [49], a lack of regional differentiation for flortaucipir is also observed; however, in that study, evaluation of off-target MAO binding via blocking studies using specific inhibitors was not performed. A decreased distribution volume under self-blocking conditions is observed and is proposed to be evidence of off-target binding; however, the study does not identify possible causes, such as binding to MAOs. As a whole, these [^18^F]flortaucipir PET studies probing potential off-target binding to MAOs have not convincingly demonstrated an association *in vivo*, prompting the current study.

In PET imaging studies using normal rodents, [^18^F]flortaucipir binding to MAO-B could not be evaluated due to low levels of MAO-B expression. In autoradiography studies of normal human brain tissue using mild aqueous wash conditions, there was minimal displacement of [^18^F]flortaucipir by MAO-B ligands (IC_50_ > 10μM). In saturation binding experiments, whereas [^18^F]THK5351 bound to a preparation of human MAO-B in microsomes with a K_d_ of 37 nM, no significant binding of [^18^F]flortaucipir to MAO-B observed; there was no difference in binding of [^18^F]flortaucipir to MAO-B microsomes than to control microsomes devoid of MAO-B or to MAO-B microsomes in the presence of 10 μM of the MAO-B inhibitor deprenyl. In another competition binding assay, flortaucipir only weakly inhibited the binding of safinamide to MAO-B with an IC_50_ of 1.3 µM. Under the same assay conditions, MAO-A inhibitors clorgyline and fluoroethyl-harmol inhibited with IC_50_ results of 3.3 µM and > 10 µM, respectively, whereas the MAO-B inhibitor deprenyl inhibited with an IC_50_ of 66 nM. Taken altogether, these data indicate that [^18^F]flortaucipir does not bind significantly to MAO-B, and any weak interaction would not be expected to impact the interpretation of [^18^F]flortaucipir PET images in humans.

The results from our saturation binding experiments disagree with results published by Vermeiren et al. [20]. Although we also observe a 2.0 nM K_d_ for [^18^F]flortaucipir binding to recombinant human MAO-A, we found no evidence of significant binding to MAO-B. Vermeiren et al. used excess non-radioactive flortaucipir to define non-specific binding [20], whereas we chose control microsomes devoid of recombinant MAO-B. We found that using excess flortaucipir to define NSB in filtration binding assays reduced [^18^F]flortaucipir non-specific binding below that of the control microsomes and produced an artifactual specific binding curve in buffer only (without any MAO or PHF). Under similar conditions as Vermeiren et al. (Supplemental Information, Fig. S2), the observed signal represents background binding of [^18^F]flortaucipir to the filter; this is then reduced by adding excess non-radioactive flortaucipir. Based on these results, we believe that the previously reported K_d_ by Vermeiren et al. may also be artifactual.

Consistent with the absence of or low interaction with MAO-B in our studies, a retrospective analysis, Hansen et al. [19] found that MAO-B inhibitors at therapeutic concentrations did not significantly affect [^18^F]flortaucipir binding in patients with Parkinson’s disease, in which MAO-B inhibitors are used to treat the disease*.* This finding is further supported by a clinical trial reported by Matthews et al. [40], in which 24 weeks of treatment with MAO-B inhibitor, rasagiline, in patients with AD did not result in changes of flortaucipir binding in cortical regions. They did report modest uniform reductions in [^18^F]flortaucipir signal in subcortical regions with high MAO-B expression, such as the striatum and nucleus accumbens [54]; however, there were no observed decreases in cortical regions with known MAO-B expression, suggesting that affinity of [^18^F]flortaucipir for MAO-B is weak and importantly much weaker than its affinity for tau [40].

Additionally, studies have demonstrated [^18^F]flortaucipir binding in the affected temporal tip in PET scans of patients with semantic variant primary progressive aphasia (svPPA), a disease characterized by TDP-43 deposition [35, 55], raising the concern that [^18^F]flortaucipir may be binding to TDP-43 or MAO-B associated with astrogliosis in these cases. However, Schaeverbeke et al. [35] found no evidence that [^18^F] flortaucipir bound to TDP-43 in autopsy tissue from svPPA patients and no effect of blocking with the MAO-B inhibitor deprenyl on [^18^F]flortaucipir binding by autoradiography [35]. Pascual et al. [55] also demonstrated that the [^18^F]flortaucipir PET scan for an svPPA patient was unaffected by pre-treatment with deprenyl, an MAO-B inhibitor. These data from Pascual and Schaeverbeke are consistent with our preclinical data and clinical studies from Hansen [19] and Matthews [40] that [^18^F]flortaucipir does not bind to MAO-B.

A possible interaction between [^18^F]flortaucipir and MAO-A would be of much less concern with respect to imaging with [^18^F]flortaucipir; unlike MAO-B, MAO-A is largely expressed in neurons, not astrocytes [56, 57], and thus should not impact PET interpretation. Due to the structural similarity between [^18^F]flortaucipir and the MAO-A ligands harmine and fluoroethyl-harmol, we also sought to characterize the binding of [^18^F]flortaucipir to MAO-A. In autoradiography experiments, displacement of [^18^F]flortaucipir binding by MAO-A ligands could be observed only using mild aqueous washing conditions and even then, only weakly (IC_50_ 0.1–1 μM). However, [^18^F]flortaucipir bound to MAO-A accounted for ~50% of binding observed, while the remaining was non-specific and non-saturable since it could not be displaced by high levels of non-radioactive flortaucipir. *In vivo* PET experiments in rats revealed no evidence of [^18^F]flortaucipir binding to MAO-A. *In vitro* studies revealed low nanomolar affinity of [^18^F]flortaucipir for MAO-A in microsomes, but kinetic binding studies indicated that the off rate of [^18^F]flortaucipir from MAO-A is much faster than [^18^F]flortaucipir from PHF tau or [^18^F]fluoroethyl-harmol from MAO-A. This fast dissociation rate from MAO-A may explain why binding of flortaucipir to MAO-A was not observed in PET imaging studies in animals, and consequently, it would not be expected that [^18^F]flortaucipir would produce images of MAO-A in humans.

In summary, autoradiography experiments showed [^18^F]flortaucipir localization or binding in tau-rich areas of AD tissue but not in normal control tissue under stringent wash conditions. In direct binding studies, flortaucipir had low nanomolar affinity toward MAO-A but with an off rate 9 times faster than for tau and PET imaging showed no significant retention consistent with binding to MAO-A in a rat model, in contrast to MAO-A ligand [^18^F]fluoroethyl-harmol. Although we could not detect any direct binding of [^18^F]flortaucipir to MAO-B or significant displacement from tissue by MAO-B ligands, a high concentration of flortaucipir blocked safinamide binding to MAO-B microsomes indicating that, at micromolar concentrations, flortaucipir may bind weakly to MAO-B *in vitro*. Overall, the *in vitro* and *in vivo* data presented here suggest that neither MAO-A nor MAO-B binding contribute significantly to [^18^F]flortaucipir PET images. The present study, together with human PET blocking studies [40, 55], clearly show that MAO binding cannot account for the flortaucipir PET signal. In conjunction with image to autopsy studies confirming the association and colocation of flortaucipir PET signal with brain NFTs, these data firmly support the interpretation of flortaucipir PET signal as an indicator of distribution and density of tau in the cortex of patients suspected of AD.

## Supplementary Information

Below is the link to the electronic supplementary material.

Supplementary file1 (PDF 189 kb)
