# Supplementary material for: Monoamine oxidase binding not expected to significantly affect [18F]flortaucipir PET interpretation
Source: Eur J Nucl Med Mol Imaging. 2022 May 21;49(11):3797–808. doi: 10.1007/s00259-022-05822-9 (PMC9399028; doi:10.1007/s00259-022-05822-9)
Supplement: Supplementary file 1 — Supplementary file1 (PDF 189 kb) [file 259_2022_5822_MOESM1_ESM.pdf]

Supplemental Information: Monoamine oxidase binding would not be expected to significantly affect [^{18}F]flortaucipir PET interpretation

Target Journal: European Journal of Nuclear Medicine and Molecular Imaging

Authors: Justin P. Wright[1], Jason R. Goodman[1], Yin-Guo Lin[1], Brian P. Lieberman[1], Jennifer Clemens[1], Luis F. Gomez[1], Qianwa Liang[1], Adam T. Hoye[1], Michael J. Pontecorvo[1], Kelly A. Conway[1]*

*Corresponding author

Affiliations: [1]Avid Radiopharmaceuticals, a wholly owned subsidiary of Eli Lilly & Company, Philadelphia, PA, USA.

First author: Justin P Wright

Corresponding author: Kelly Conway

3711 Market Street

Seventh Fl Philadelphia, PA 19104, USA

conway@avidrp.com

Supplemental Methods

Quadrupole Time-of-Flight Mass Spectrometer (QTOF-MS)

Samples (15 µL) were injected on a Waters Acquity UPLC system equipped with a Waters Acquity T3 HSS, 2.1 x 50mm, 1.8 µm column at a mobile phase flow rate of 0.5 mL/min, and (MS) response was monitored with a Waters Xevo QTOF scanning 50-1200 Da. For each sample analysis, the column was equilibrated with 95:5 water with 0.1% formic acid (Mobile Phase A): acetonitrile with 0.1% formic acid (Mobile Phase B) for 0.25 minutes. Following injection, the gradient was changed to 70:30 Mobile Phase B: Mobile Phase A in a linear fashion over 1.75 minutes, and then to 100% Mobile Phase B over 0.2 minutes, held at 100% Mobile Phase B for 0.8 minutes, followed by 95:5 Mobile Phase A:Mobile Phase B over 0.1 minutes and held for 0.4 minutes. Masslynx QuanLynx™ software was used to analyze MS data. An internal standard was added to all samples to correct for instrument and sample processing variability.

Supplemental Results

[^{18}F]Flortaucipir autoradiography in rat brain tissue using mild wash conditions

Experimental conditions as described in the Materials and Methods section of the manuscript "[^{18}F]Flortaucipir Autoradiography: Comparison of Stringent and Mild Wash Conditions". Under mild wash conditions, high background ARG signal is seen in rat brain tissue samples (male wild-type Sprague Dawley). The MAO-B inhibitor deprenyl (1 µM) has no blocking effect on [^{18}F]flortaucipir ARG signal. The MAO-A/B inhibitor pargyline (10 µM), MAO-A inhibitor, clorgyline (1 µM), and flortaucipir (1 µM) partially block [^{18}F]flortaucipir ARG signal.

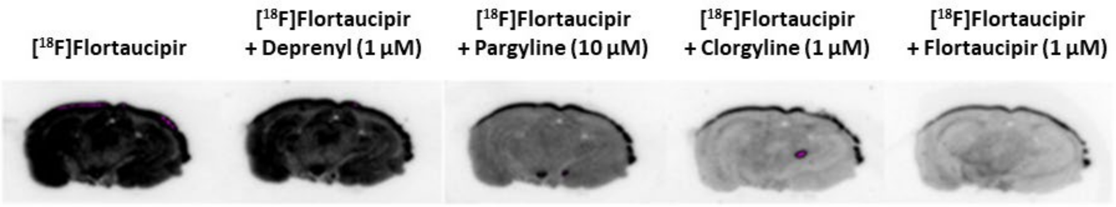

Figure S1. Autoradiography of [^{18}F]flortaucipir in rat brain tissue with blocking by MAO-A/MAO-B inhibitors and flortaucipir.

Possible artifactual saturation binding curve

Saturation binding for [^{18}F]flortaucipir against recombinant monoamine oxidase-B (MAO-B) when using 1 µM [^{18}F]flortaucipir to define non-specific binding (A) generates an artifactual [^{18}F]flortaucipir:MAO-B saturation binding curve with a K_d of 28 nM by artificially reducing the [^{18}F]flortaucipir nonspecific binding below that of the control microsomes. A similar artifactual binding curve with a K_d of 16 nM is generated for [^{18}F]flortaucipir curves run in binding buffer only, in which 10 µM flortaucipir is used to define NSB (B). This signal represents background binding of [^{18}F]flortaucipir to the filter, which can be reduced by adding saturating amounts of non-radioactive flortaucipir.

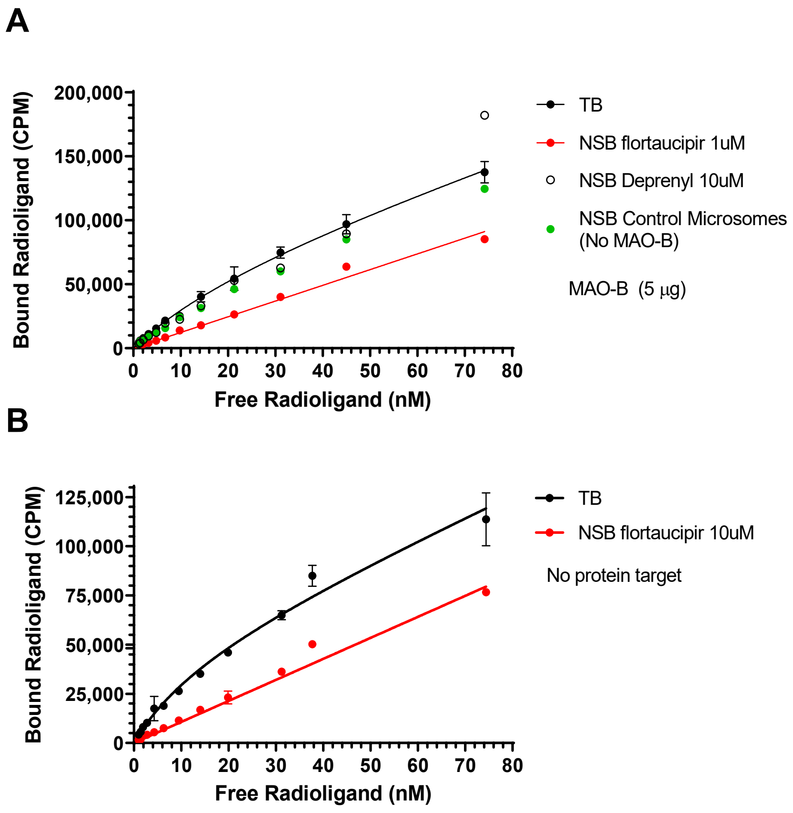

Figure S2. Artifactual [^{18}F]flortaucipir binding curves generated by using high concentration of flortaucipir to define nonspecific binding.

Abbreviations: CPM, counts per minutes; MAO, monoamine oxidase; NSB, Non-specific binding; TB, Total Binding.
